# Pulmonary pleomorphic carcinoma with multiple metastases to the right posterior knee complicated by paraneoplastic hypercalcemia

**DOI:** 10.3892/ol.2013.1742

**Published:** 2013-12-09

**Authors:** PENG-FEI LI, CHENG-HSIANG LO, SHAN-HAN YANG, PING-YING CHUNG, CHING-LIANG HO

**Affiliations:** 1 Department of Internal Medicine, Tri-Service General Hospital, National Defense Medical Center, Neihu 114, Taipei, Taiwan, R.O.C.; 2 Department of Radiation Oncology, Tri-Service General Hospital, National Defense Medical Center, Neihu 114, Taipei, Taiwan, R.O.C.

**Keywords:** pulmonary pleomorphic carcinoma, right posterior knee mass, paraneoplastic hypercalcemia

## Abstract

In this report, we describe the case of a 46-year-old male who presented with a three-month history of progressive intermittent pain over the right posterior knee. Magnetic resonance imaging showed soft tissue masses over the right popliteal fossa. Surgery was performed, and histological examination revealed the mass to be a sarcomatoid carcinoma of poor differentiation. Fluorodeoxyglucose (FDG)-positron emission tomography showed FDG uptake in the lungs and in the right para-aortic and popliteal regions. On the basis of the morphological and immunohistochemical features of the specimens, the patient’s condition was diagnosed as a pulmonary pleomorphic carcinoma with multiple metastases. Systemic chemotherapy was initiated with paclitaxel and cisplatin. The patient then developed paraneoplastic hypercalcemia and ultimately succumbed to healthcare-acquired pneumonia. The results of this rare case indicate that pulmonary pleomorphic carcinomas respond poorly to combination chemotherapy with paclitaxel and cisplatin. The firm mass in the popliteal fossa that was situated behind the knee was considered to be a Baker cyst; however, the possibility of malignant metastatic sarcomas, such as pulmonary sarcomatoid carcinoma, should be considered in the differential diagnosis. In conclusion, we emphasize that pretherapeutic examinations should be the basis for the diagnosis of a mass lesion at either an unusual or usual site.

## Introduction

Pulmonary sarcomatoid carcinomas (SCs) are a heterogeneous group of non-small cell lung carcinomas with a rare histological subtype, and they have been reported to have a poor prognosis (1–3,5). The overall survival rate of pulmonary SC is significantly lower than that for other non-small cell lung carcinomas (1,2,10). The different types of pulmonary SC include pleomorphic carcinoma (PC), spindle cell carcinoma, giant cell carcinoma, carcinosarcoma and pulmonary blastoma (1). The diagnosis of pulmonary SC is difficult using small biopsy specimens and typically requires resection specimens (1). Pulmonary SC predominantly occurs in males with a mean age of 60 years at diagnosis and who are heavy smokers (1,2,4,6). PC, the most common subtype of pulmonary SC according to the World Health Organization classification of histological cancer, accounts for 0.3% of all invasive lung malignancies of high grade with an aggressive clinical course. The mean or median survival time of patients with PC ranges from five to 35 months (1,2). This subtype of SC tumor occurs more frequently in the thorax than do true sarcomas. Written informed consent was obtained from the patient’s family.

## Case report

**Figure 1:**
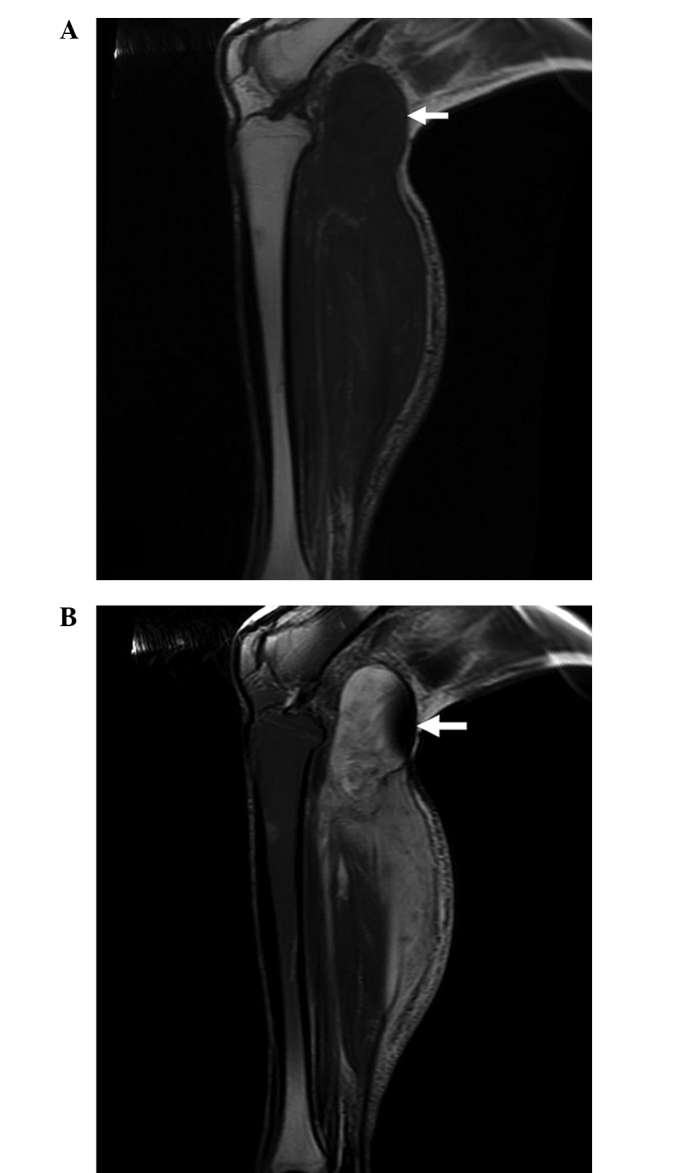
MRI of the sagittal plane of the patient’s right leg showed one large lobulated cystic mass (arrow) filled with debris or tissue thickening, measuring ~5.9×5.4×8.6 cm over the popliteal fossa. (A) T1-weighted MRI showed low signal. (B) T2-weighted MRI showed high signal. MRI, magnetic resonance imaging.

**Figure 2:**
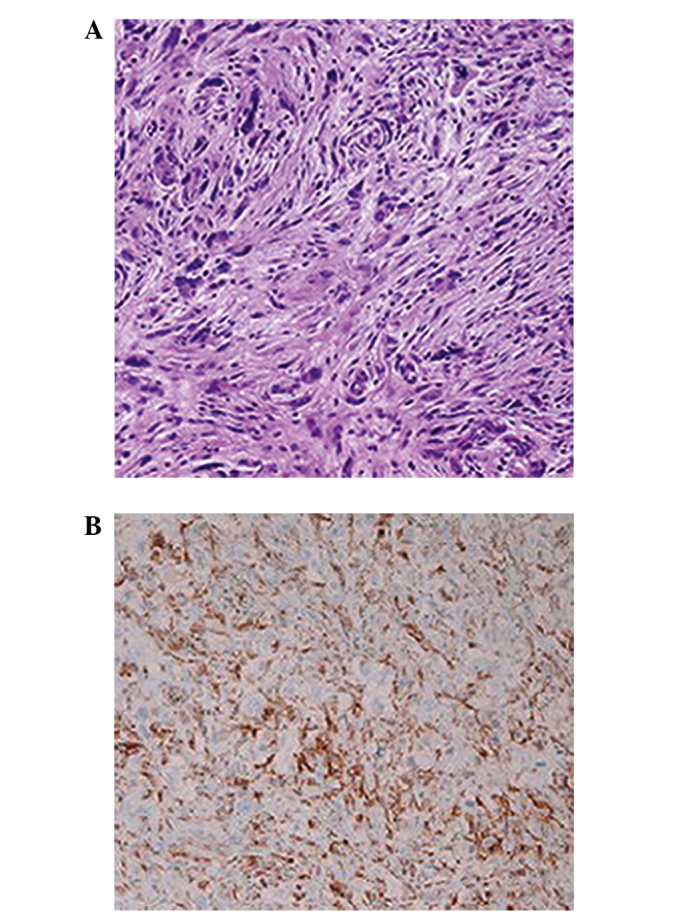
(A) Histopathological features of the specimen showing a poorly differentiated carcinoma, with sarcomatoid changes characterized by a solid and focal individual tumor growth pattern composed of marked pleomorphic tumor cells. (B) Vimentin staining was positive. Immunostaining; magnification, ×100.

A previously healthy 46-year-old male presented with progressive swelling and mass formation in the right popliteal region. The patient had a three-month history of progressive post-popliteal soreness and tightness behind the knee, particularly when the knee was extended or fully flexed. The patient was subsequently admitted to the Neurology Department of Tri-Service General Hospital (Tapei, Taiwan). In March 2012, magnetic resonance imaging (MRI) showed a large lobulated cystic mass filled with debris or tissue thickening, measuring approximately 5.9×5.4×8.6 cm over the popliteal fossa (Fig. 1). The complete blood count results were as follows: White blood cell count, 14.69×10^9^/l; hemoglobin count, 96 g/l; platelet count, 240×10^9^/l; and serum calcium, 13.9 mg/dl. The patient immediately underwent surgery for resolution of the neurological symptoms. Pathological evaluation of the popliteal mass showed a poorly differentiated carcinoma, with sarcomatoid changes characterized by a solid and focal individual tumor composed of marked pleomorphic tumor cells of the soft tissue (Fig. 2A). Immunohistochemistry showed that the popliteal mass was positive for Ki67, p53, p63 and vimentin (Fig. 2B). The morphological and immunohistochemical features were comparable with those of metastatic SC. Fluorodeoxyglucose (FDG)-positron emission tomography (PET) was performed immediately, and FDG uptake was observed in the lungs and the right para-aortic and popliteal regions. Based on these findings, the final histological diagnosis was a pulmonary metastatic squamous cell carcinoma with sarcomatoid changes and a pleomorphic subtype. Systemic chemotherapy was initiated following diagnosis; it consisted of a combination of cisplatin [(100 mg intravenous (i.v.) on day one)] and paclitaxel (115 mg i.v. on day one and 130 mg (i.v.) on day eight) for eight days. However, the patient developed progressive consciousness disturbance and shortness of breath following chemotherapy. The serum levels of calcium and parathyroid hormone-related protein were 18.9 mg/dl and 3.3 pmol/l, respectively, and PET revealed no bony metastasis. Hypercalcemia occurred as a paraneoplastic syndrome of pulmonary SC. The patient was treated with hydration, urgent hemodialysis, i.v calcitonin and bisphosphonates. Due to the weak condition of the patient, anticancer treatment was discontinued. However, dyspnea developed and the patient developed pneumonia four days after the first course of chemotherapy. The patient’s family refused further treatment and intervention due to the poor prognosis. The patient succumbed to healthcare-acquired pneumonia in May 2012 with severe sepsis due to a *Pneumocystis jiroveci* infection.

## Discussion

The present report describes a case of pulmonary PC complicated by a soft tissue mass in the right posterior knee with progressive post-popliteal soreness and stiffness. Pulmonary PCs are rare, accounting for 0.3% of all invasive lung malignancies, and they frequently present as large tumors with a mean size of 5–8 cm (range, 1–28 cm) (1,2). However, in the present case, the patient presented with a rare clinical profile of multiple nodules in the lungs and metastases to the right para-aortic and popliteal regions, rather than a single solid mass in the lung. On the basis of fluid distention of the gastrocnemio-semimembranosus bursa, the unilateral popliteal mass without redness, local heat or trauma history was first thought to be a Baker cyst, also termed a popliteal cyst. However, the possibility of a malignant neoplasm should not be ruled out despite an MRI showing a single large lobulated cystic mass that is considered to be benign. The present case highlights the importance of a correct diagnosis. Pretherapeutic examinations, such as chest radiography, abdominal sonography or computed tomography, should be the basis for the diagnosis of a mass lesion in an unusual or usual site, as observed in the present case of a popliteal mass lesion.

Pathologically, the majority of these tumors can be classified using light microscopy alone. The diagnosis of these tumors requires a resected specimen, largely owing to the histological heterogeneity and pleomorphism of the tumor. Small biopsy specimens with adequate cytological material presented with loose clusters of poorly differentiated epithelial cells, giant cells, malignant spindle cells and a necrotic background with neutrophils and lymphocytes, which are highly indicative of PC (6). The spindle cells and giant cells of PC usually stain with epithelial markers such as pancytokeratin (i.e. AE1/AE3), CAM 5.2, CK18 and EMA; however, in a small percentage of cases, the staining results may be negative. In the present case report, immunohistochemistry showed that the mass was positive for Ki67, p53, p63 and vimentin (15). The morphological and immunohistochemical features were compatible with those of metastatic SC.

Systemic chemotherapy with a single course of paclitaxel and cisplatin was unsuccessful. The patient developed a paraneoplastic syndrome consisting of hypercalcemia, and eventually acquired pneumonia. A previous case report indicated that pulmonary PC responds well to a combination of gemcitabine and docetaxel (7); however, other studies support the view that pulmonary PC responds poorly to chemotherapy and targeted therapy (8,11–14). To date, no standard chemotherapy regimen for pulmonary PC has been established, and our case indicates that pulmonary PC responds poorly to combination chemotherapy with paclitaxel and cisplatin. In our case, hypercalcemia associated with pulmonary PC showed aggressive disease progression and a poor prognosis. The addition of combination therapies (i.v. calcitonin and bisphosphonates) to anticancer drugs for paraneoplastic hypercalcaemia may be beneficial for improving patient prognosis.

In conclusion, pulmonary sarcomatoid neoplasms are rare, and we report here the first case of pulmonary PC with multiple metastases to the right posterior knee. The present case indicates that pulmonary PC with paraneoplastic hypercalcemia responds poorly to combination chemotherapy with paclitaxel and cisplatin. The addition of combination therapies, such as i.v. calcitonin and bisphosphonates, to anticancer drugs may be beneficial in cases of paraneoplastic hypercalcemia, which is associated with aggressive disease progression and a poor prognosis. Notably, we emphasize that pretherapeutic examinations should be the basis for the diagnosis of a mass lesion at either an unusual or usual site, such as the popliteal mass lesion presented in this case report.
